# A Dictionary of Practical Medicine

**Published:** 1891-05

**Authors:** 


A Dictionary of Practical Medicine. By various writers. Edited by J. Kingston Fowler, M. A., M. D., Fellow of the Royal College of Physicians; Senior Assistant to the Middlesex Hospital, and Lecturer on Pathological Anatomy in the Medical School; Senior Assistant Physician to the Hospital for Consumption and Diseases of the Chest, Brompton. 8vo; pp. xxvi.—942. Philadelphia: P. Blakiston, Son & Co. 1890. Buffalo: Peter Paul & Bro.

Since a reference book is a necessity, it is well to have a good one. Not every physician feels that he can equip his book-shelves with the larger dictionaries of medicine — the encyclopedias — so there must needs be smaller works. There is no danger of our being misunderstood when we admit that reference books are needful. Every physician must refresh his memory on some point, especially when writing, that general treatises either do not supply at all, or else bury the information sought so deeply that it is difficult to find it. The book before us will be found a great help to such, as well as to the young physician who does not yet feel able to purchase a complete library. The names of the contributors are a guaranty of accuracy, and we hesitate not to pronounce this book one of the best of its kind,— safe, advanced, and yet within the reach of the most modest purse.

